# Association of physical functional activity impairment with severity of sarcopenic obesity: findings from National Health and Nutrition Examination Survey

**DOI:** 10.1038/s41598-024-54102-z

**Published:** 2024-02-15

**Authors:** Shih-Wei Huang, Yu-Hao Lee, Chun-De Liao, Reuben Escorpizo, Tsan-Hon Liou, Hui-Wen Lin

**Affiliations:** 1 Department of Physical Medicine and Rehabilitation, Shuang Ho Hospital, Taipei Medical University, Taipei, Taiwan; 2 Department of Physical Medicine and Rehabilitation, School of Medicine, College of Medicine, Taipei Medical University, Taipei, Taiwan; 3 International Ph.D. Program in Gerontology and Long-Term Care, College of Nursing, Taipei Medical University, Taipei, Taiwan; 4 Department of Rehabilitation and Movement Science, College of Nursing and Health Sciences, University of Vermont, Burlington, VT USA; 5 Swiss Paraplegic Research, Nottwil, Switzerland; 6 Department of Mathematics, Soochow University, Taipei, Taiwan

**Keywords:** Sarcopenia, Obesity, Sarcopenic obesity, Physical function, Impairment, Obesity, Disability

## Abstract

We aim to clarify the relationship between low skeletal muscle mass and varying levels of adiposity and to identify the types of physical function impairments associated with sarcopenic obesity (SO). This study examined cross-sectional data from the National Health and Nutrition Examination Survey with whole-body dual-energy X-ray absorptiometry (DXA) scans. The data included age, gender, DXA-assessed body composition, and physical functional activity with performing daily tasks by questionnaire. We subdivided the data by body composition into a non-SO group and a SO group (ASMI 0–49.99% and FMI of 50–100%), after which the SO data were subdivided into three classes. A higher class indicated higher adiposity and lower muscle mass. The physical function impairment of the two groups was compared. Our study examined 7161 individuals, of which 4907 did not have SO and 2254 had SO, and their data were further divided into three classes (i.e., class I, 826 individuals; class II, 1300 individuals; and class III, 128 individuals). Significant differences in demographics and DXA parameters were identified between the non-SO and SO groups (*P* < 0.001); the individuals with SO were older, included more women, and exhibited high adiposity and less lean muscle mass. The individuals with class III SO exhibited greater differences and reported more difficulty in performing daily activities. The individuals with class III SO exhibited the most severe physical function impairment. Our study highlights the considerable difficulties encountered by individuals with SO in performing daily activities. Given this finding, customized rehabilitation strategies should be implemented to improve the quality of life of individuals with SO.

## Introduction

Sarcopenia, characterized by the age-related, progressive, and generalized deterioration of the skeletal muscle, encompasses the reduction of muscle mass, strength, and physical performance^1^. In the Asian population, the prevalence of sarcopenia, as determined by the diagnostic criteria of the Asia Working Group for Sarcopenia 2014, ranges from 7.3 to 12%^2^. Increasing research has emphasized the adverse health implications of sarcopenia, including falls, functional decline, hospitalization, frailty, escalated health-care expenditure, and mortality^3^. Aging-related changes in body composition lead not only to reduced muscle mass but also to an increase in the body fat mass. These changes can cause obesity and increase the risks of metabolic and cardiovascular diseases. Sarcopenic obesity (SO) is characterized by both reduced muscle mass and increased adiposity^4,5^. Both sarcopenia and obesity have common inflammatory pathways, and their combined negative effects can exacerbate functional deterioration. SO contributes to the risks of metabolic syndrome, physical disability, and mortality in older adults^6^. To protect public health, effectively preventing and addressing SO are crucial.

In 2001, the International Classification of Functioning, Disability, and Health (ICF) was formally endorsed by the World Health Organization (WHO). The ICF framework is used to catalogue various aspects of health and health-related domains, providing unified terminology for describing and quantifying health and disability. In addition to health and functioning, this framework can be used by professionals to evaluate the activities and environmental factors that influence an individual’s ability to fully participate in society. Under the ICF framework, the execution of a task or action by an individual is termed as an “activity,” whereas their involvement in a life situation is defined as “participation.” The ICF framework can be used to organize and define data regarding health-related topics, the deterioration of body functions or structures (e.g., cognitive ability and muscle strength), activity-related constraints, restrictions to participation, and pertinent environmental effects^7^.

An expanding body of evidence is suggesting that the simultaneous presence of high adiposity and low muscle mass (HA–LM) is linked to increased health risks of various conditions^8–10^. A recent investigation revealed that sarcopenia is characterized by lower-limb muscle atrophy, and in older women, obesity is linked to diminished strength in both upper and lower limbs^11^. Despite the growing interest in understanding this body composition phenotype (i.e., HA–LM), the types of physical and functional impairments affecting individuals with HA–LM are understudied. Therefore, we conducted this study to identify the relationship between reduced skeletal muscle mass and different degrees of adiposity, as well as to pinpoint the specific types of physical function impairments linked to sarcopenic obesity (SO).

## Methods

### Study design and database

The present cross-sectional study examined secondary data obtained from the NHANES database. The NHANES, a program launched by the National Center for Health Statistics (NCHS), Centers for Disease Control and Prevention (CDC), the United States, is an ongoing series of surveys that combine interviews and examinations to assess the health and nutritional status of adults and children. The NHANES, conducted using a complex multistage design, obtains and analyzes data that are representative of the noninstitutionalized population of the United States. The NCHS allows researchers to use NHANES data, which are released for research purposes. NHANES participants undergo a household interview and are invited to undergo a comprehensive examination at a mobile examination center, which comprises a physical examination, specialized measurements, and laboratory tests. Such evaluation of individuals in the NHANES program produces data that are reliable, multidimensional, and comparable to those obtained by a population-level assessment^12^. The NHANES program has been reviewed and approved by the Research Ethics Review Board of the NCHS, and written informed consent was obtained from all survey participants. Consequently, no additional ethical approval or informed consent is required to analyze the secondary data in the present study. Furthermore, all NHANES data released by the NCHS have been deidentified to ensure anonymity during data analysis.

### Study participants

**Figure 1:**
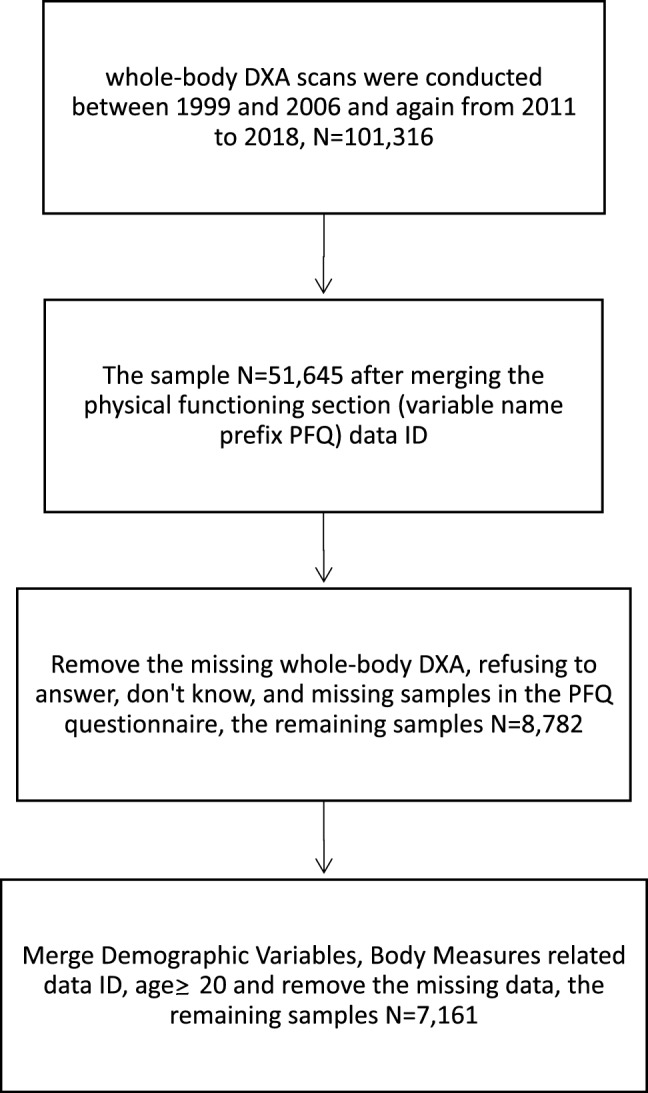
Flow diagram of study.

This study utilized cross-sectional data collected from the NHANES and whole-body dual-energy-X-ray-absorptiometry (DXA) scans that were taken between 1999 and 2006 and between 2011 and 2018; the examined data included age, gender, and DXA-assessed body composition. The NHANES releases DXA data sets on the CDC website (http://www.cdc.gov/nchs/about/major/nhanes/dxx/dxa.htm). In the NHANES, whole-body DXA scans were obtained using a QDR 4500A fan beam densitometer (default configuration, software version 12.1; Hologic). In relation to safety considerations, it is noteworthy that the radiation exposure associated with DXA whole body scans is exceptionally minimal, measuring at less than 10 microsieverts. To ensure the reliability of the gathered data, a stringent quality control regimen was upheld during both the DXA data collection and scan analysis processes, incorporating a rigorous schedule for phantom scanning. The present study focused on 7161 adults (aged > 20 years) with available data on DXA-assessed body composition. Individuals were excluded from our analysis if their stated weight exceeded the maximum of 136 kg or if their recorded height exceeded the maximum of 196 cm, as indicated in the DXA scan table. Female NHANES participants were excluded from the DXA assessment if they tested positive for pregnancy or self-reported being pregnant at the time of examination. The study flowchart is presented in Fig. 1.

The demographic information examined in the present study comprised age, gender, body weight, and body height. Data pertaining to body measurements were acquired within the confines of the Mobile Examination Center (MEC) under the supervision of proficient health technicians. Throughout the body measures examination, a dedicated recorder provided assistance to the health technician. The specific protocol employed for the body measures examination was determined based on the participant's age at the time of the screening interview. The body mass index (BMI) of an individual was determined by measuring their standing height (in meters) and body weight (in kilograms). The examined body composition variables were the whole-body DXA measurements of fat mass and lean soft tissue (LST). DXA-assessed LST comprises all fat-free mass components, except for the bone mineral content. On the basis of these whole-body measures, the fat mass index (FMI; formula = fat mass/height^2^) and appendicular skeletal muscle mass index (ASMI; formula = appendicular lean mass/height^2^) were calculated^13–16^. Based on objective body composition assessment tool in NHANES database and stratification different degrees of SO severity. In the present study, the body composition phenotype was classified as per the framework used by Prado et al.^17^, and individuals with an ASMI of 0–49.99% and FMI of 50–100% were defined as having SO. For individuals with SO, SO was further classified as class I SO (ASMI of 0–49.99% with FMI of 50–59.99% or ASMI of 40–49.99% with FMI of 60–100%), class II SO (ASMI of 0–39.99% with FMI of 60–79.99% or ASMI of 20–39.99% with FMI of 80–100%), and class III SO (ASMI of 0–19.99% with FMI of 80–100%). A higher class indicates higher adiposity with lower muscle mass.

### Physical disability and functional impairment

The primary outcome variables in the present study were derived from the Physical Functioning Questionnaire, which is used to obtain self-reported data on functional limitations^18–20^. We focused on 14 activities that embodied the concepts of physical activities and participation restriction as defined in the ICF framework. These activities can be classified as (1) Activities of daily living (disability): attending social events, dressing oneself, getting in and out of bed, attending a movie screening or an event, grasping or holding small objects, engaging in home-based leisure activities, lifting or carrying items, performing household chores, preparing meals, and (2) activities assessing physical abilities such as strength, mobility, balance (functional impairments): standing up from an armless chair, standing for extended periods, sitting for extended periods, walking a quarter mile, and climbing 10 steps. NHANES participants were asked to evaluate the level of difficulty they experienced when performing the aforementioned activities independently without any special equipment. The response options were “no difficulty,” “some difficulty,” “considerable difficulty,” and “unable to perform the task.” If a participant did not perform the activity, declined to answer, or was unsure if they had performed the activity, their data were considered missing and excluded from the main data set. To identify limitations, responses were coded as follows: “no difficulty” = 1, “some difficulty” = 2, “considerable difficulty” = 3, and “unable to perform the task” = 4. We then calculated each individual’s average score for all responses; those with a score of 1 were regarded as having no physical functional activity limitations, whereas those with a score of ≥ 2 were regarded as having physical functional activity limitations in at least one activity.

### Statistical analysis

The differences between individuals with and without SO were determined through Student’s *t* test for continuous variables and chi-square tests for categorical variables. If these continuous variables were not normality and homogeneity of variance distributed, we will use non-parametric alternatives such as the Mann–Whitney U test for continuous variables. For categorical variables, if the expected frequencies in the chi-square tests are too low, we will use Fisher's exact test as an alternative. Additionally, analysis of variance (ANOVA) was performed to compare continuous variables such as age, BMI, and body composition parameters between the individuals without SO and those with varying levels of SO. For understanding different presentation of SO among different genders, we separated male and female genders for further analysis. Chi-square tests were conducted for categorical variables such as gender. A Mann–Whitney U test was performed to compare the individuals with and without SO in terms of the average (standard deviation) of the ranked difficulty of ICF concept–related physical functional activities. Kruskal Wallis Test was performed to compare the average difficulty of performing physical functional activities between the individuals with varying levels of SO and those without SO. Multivariate model was analyzed for activity limitations of SO and non-SO participants with different classification of SO after adjusted gender and age. All data were analyzed using statistical software SPSS (version 22; SPSS, Chicago, IL, USA) and SAS (version 9.1.3; SAS Institute, Cary, NC, USA) with obtained copyright license with significance level (alpha) of 0.05 for all statistical tests.

## Results

**Figure 2:**
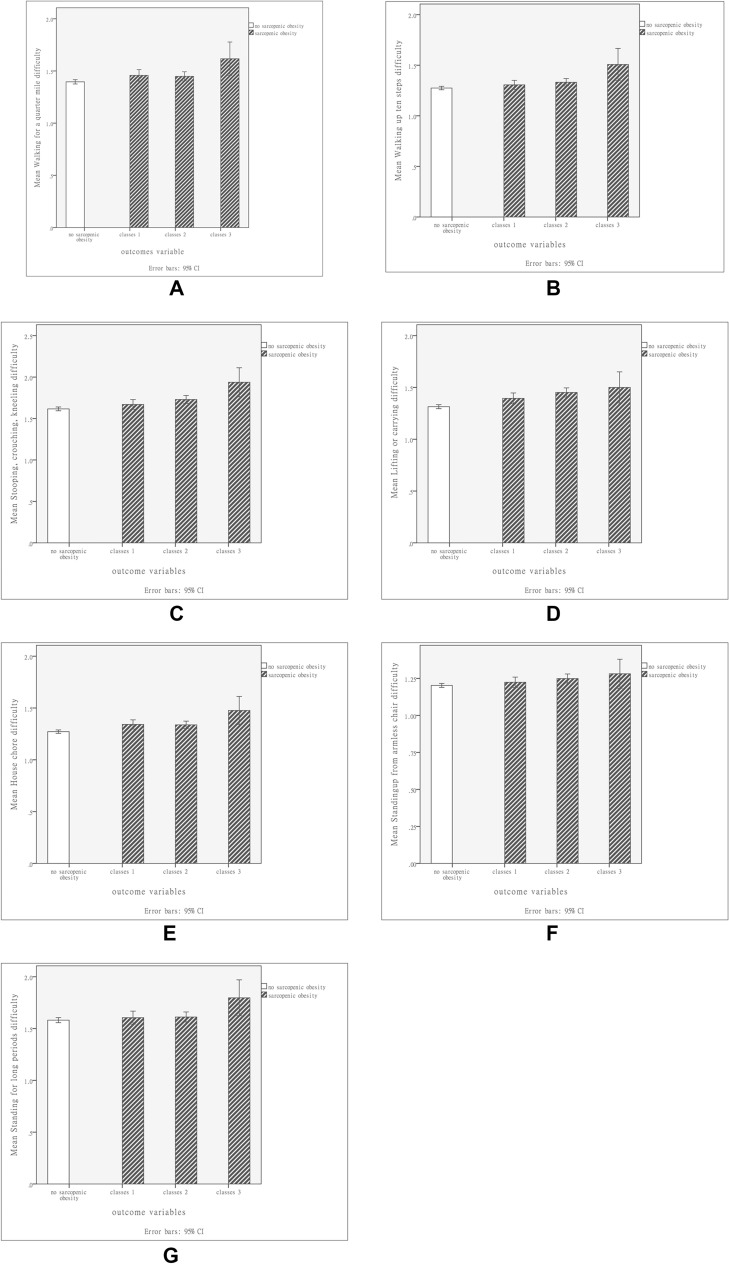
Physical activity limitations of study groups with respect to (**A**) walking a quarter mile; (**B**) climbing 10 steps; (**C**) stooping, crouching, and kneeling; (**D**) lifting or carrying; (**E**) performing household chores; (**F**) standing up from an armless chair; and (**G**) standing for long periods.

In total, 7161 individuals were selected and enrolled into the present study. Among them, 4907 did not have SO (non-SO group), and 2254 had SO (SO group). In the SO group, 826, 1300, and 128 were classified as having class I, class II, and class III SO. Table 1 lists the differences in demographic data and DXA-related parameters between the non-SO group and SO group. Relative to the individuals in the non-SO group, those in the SO group were older; exhibited higher total fat, fat percentage, FMI, and BMI; included more women; and exhibited lower levels of lean muscle mass and ASMI (*P* < 0.001 for all variables). Table 2 lists the differences in demographic and body composition variables between the individuals without SO and those with varying levels of SO. Compared with the non-SO group and the individuals with other levels of SO, those with class III SO were older; included more women; and exhibited higher total fat, fat percentage, and FMI (*P* < 0.001 for all variables). The analysis of different genders with SO and non-SO were presented as Table S1, S2, S3 and S4. In addition, the individuals with class III SO exhibited lower levels of lean muscle mass, total bone mineral density, and ASMI (*P* < 0.001 for all variables). Regarding daily activities, the individuals with SO reported more difficulty in walking a quarter mile (*P* = 0.003); Walking 10 steps (*P* = 0.001); stooping, crouching, and kneeling (*P* < 0.001); performing household chores (*P* < 0.001); standing up from an armless chair (*P* = 0.005); and reaching overhead (*P* = 0.032) relative to the individuals without SO (Table 3). After adjusted gender and age, the multi-variate model presented SO participants with functional activities limitations about walking a quarter mile (*P* < 0.001); walking up 10 steps (*P* < 0.001); stooping, crouching, and kneeling (*P* = 0.007); performing household chores (*P* = 0.004); and standing for long periods (*P* < 0.001) relative to the individuals without SO (Table 4). Among the individuals with varying levels of SO, those with class III SO reported more difficulty in walking a quarter mile (*P* = 0.002); walking up 10 steps (*P* = 0.001); stooping, crouching, and kneeling (*P* < 0.001); lifting or carrying (*P* < 0.001); performing household chores (*P* < 0.001); standing up from an armless chair (*P* = 0.017); standing for long periods (*P* = 0.039), and leisure activity at home (*P* = 0.026) (Table 5). Figure 2 presents the physical functional activity limitations of the individuals with varying levels of SO, and it reveals that those with class III SO found it more difficult to perform daily activities. For different classification of SO, the multi-variate model with adjusted of age and sex presented functional activities limitations about walking a quarter mile (*P* < 0.001); walking up 10 steps (*P* < 0.001); stooping, crouching, and kneeling (*P* = 0.003); performing household chores (*P* = 0.002); and standing for long periods (*P* < 0.001) relative to the individuals without SO (Table 6).

**Table 1:** Demographic and body composition characteristics of sarcopenic obesity (SO) and non-sarcopenic obesity (non-SO) participants.

	Non-SO (N = 4907)		SO (N = 2254)		*P* value
	Mean	SD	Mean	SD	
Age	57.07	16.84	63.75	14.59	< 0.001
Sex					< 0.001
Male (N, %)	3287 (94.8%)		179 (5.2%)		
Female (N, %)	1620 (43.8%)		2075 (56.2%)		
Total area (cm^2^)	2130.38	256.13	1857.07	188.80	< 0.001
Total BMC (g/cm^2^)	1.13	0.13	1.02	0.12	< 0.001
Total fat (g)	28,631.63	12,945.24	29,817.25	6323.49	< 0.001
Total lean excl BMC (g)	53,723.77	11,443.32	39,312.55	5553.35	< 0.001
Total lean + fat (g)	84,781.46	21,138.08	71,033.77	10,356.56	< 0.001
Total percent fat	32.67	8.35	41.76	4.43	< 0.001
Weight (kg)	84.23	21.08	70.53	10.36	< 0.001
Standing height (cm)	169.53	9.54	159.94	7.79	< 0.001
BMI (kg/m^2^)	29.29	7.11	27.54	3.41	< 0.001
ASMI	8.09	1.52	6.21	0.68	< 0.001
FMI	10.08	4.88	11.68	2.46	< 0.001

Chi-square analysis was used for comparing categorial variables between non-SO and SO groups with different classification.

ANOVA was used for comparing continuous variables between non-SO and SO groups with different classification.

*BMC* bone mineral density, *BMI* body mass index, *ASMI* appendicular skeletal muscle mass index, *FMI* fat mass index.

**Table 2:** Demographic and body composition characteristics of sarcopenic obesity (SO) and non-sarcopenic obesity (non-SO) participants with different severity.

	Non-SO (N = 4907)		Classes 1-SO (N = 826)		Classes 2-SO (N = 1300)		Classes 3-SO (N = 128)		*P* value
	Mean	SD	Mean	SD	Mean	SD	Mean	SD	
Age	57.07	16.84	61.71	15.64	64.57	13.93	68.59	12.05	< 0.001
Sex									< 0.001
Male (N, %)	3287 (94.8%)		137 (4.0%)		42 (1.2%)		0 (0%)		
Female (N, %)	1620 (68.5%)		689 (18.6%)		1258 (34%)		128 (1.8%)		
Total area (cm^2^)	2130.38	256.13	1903.12	209.80	1830.88	171.90	1825.98	149.61	< 0.001
Total BMD (g/cm^2^)	1.13	0.13	1.04	0.12	1.01	0.12	.98	0.10	< 0.001
Total fat (g)	28,631.63	12,945.24	29,257.49	7798.22	29,800.92	5267.46	33,595.15	3755.53	< 0.001
Total lean excl BMC (g)	53,723.77	11,443.32	41,774.42	6271.21	38,121.27	4549.22	35,524.68	3396.54	< 0.001
Total lean + fat (g)	84,781.46	21,138.08	73,031.62	12,468.30	69,776.31	8872.43	70,912.44	6750.85	< 0.001
Total percent fat	32.67	8.35	39.64	4.99	42.56	3.37	47.34	1.99	< 0.001
Weight (kg)	84.23	21.08	72.53	12.48	69.27	8.86	70.35	6.76	< 0.001
Standing height (cm)	169.53	9.54	161.58	8.39	159.08	7.34	158.12	6.17	< 0.001
BMI (kg/m^2^)	29.29	7.11	27.74	4.25	27.36	2.89	28.08	1.60	< 0.001
ASMI	8.09	1.52	6.59	0.70	6.04	0.55	5.48	0.34	< 0.001
FMI	10.08	4.88	11.24	3.06	11.79	2.01	13.41	1.00	< 0.001

Chi-square analysis was used for comparing categorial variables between non-SO and SO groups.

Independent *t* test was used for comparing continuous variables between non-SO and SO groups.

*BMC* bone mineral density, *BMI* body mass index, *ASMI* appendicular skeletal muscle mass index, *FMI* fat mass index.

**Table 3:** Activity limitations of sarcopenic obesity (SO) and non-sarcopenic obesity (non-SO) participants.

Difficulty activities	Non-SO (N = 4907)		SO (N = 2254)		*P* value
	Mean	SD	Mean	SD	
Walking for a quarter mile	1.40	.748	1.46	.814	0.003*
Walking up ten steps	1.27	.618	1.33	.684	0.001*
Stooping, crouching, kneeling	1.62	.819	1.72	.890	< 0.001*
Lifting or carrying	1.31	.695	1.43	.799	< 0.001*
House chore	1.27	.592	1.35	.659	< 0.001*
Preparing meals	1.11	.420	1.10	.418	0.884
Standing up from armless chair	1.20	.493	1.24	.544	0.005*
Getting in and out of bed	1.17	.425	1.18	.441	0.954
Standing for long periods	1.58	.881	1.62	.909	0.122
Sitting for long periods	1.33	.641	1.34	.643	0.574
Reaching up over head	1.21	.541	1.25	.581	0.032*
Grasp/holding small objects	1.17	.449	1.19	.480	0.128
Going out to movies, events	1.21	.543	1.23	.571	0.242
Attending social event	1.20	.536	1.20	.539	0.407
Leisure activity at home	1.09	.335	1.08	.345	0.181

Mann–Whitney U test was used for comparing continuous variables between non-SO and SO groups.

*P< 0.05.

**Table 4:** Multivariate model for activity limitations of sarcopenic obesity (SO) and non-sarcopenic obesity (non-SO) participants after adjusted gender and age.

Difficulty activities	Mean square	F	*P* value
Walking for a quarter mile	9.472	16.309	< 0.001*
Walking up ten steps	7.565	18.911	< 0.001*
Stooping, crouching, kneeling	5.035	7.239	0.007*
Lifting or carrying	1.870	3.595	0.058
House chore	3.093	8.399	0.004*
Preparing meals	0.225	1.279	0.258
Standing up from armless chair	0.686	2.669	0.102
Getting in and out of bed	0.107	0.585	0.445
Standing for long periods	15.190	19.460	< 0.001*
Sitting for long periods	1.105	2.735	0.098
Reaching up over head	0.092	0.300	0.584
Grasp/holding small objects	0.046	0.217	0.641
Going out to movies, events	0.653	2.154	0.142
Attending social event	0.463	1.620	0.203
Leisure activity at home	0.105	0.917	0.338

*P < 0.05.

**Table 5:** Activity limitations of sarcopenic obesity (SO) and non-sarcopenic obesity (non-SO) participants with different classification.

Difficulty activities	Non-SO (N = 4907)		classes 1-SO (N = 826)		classes 2-SO(N = 1300)		classes 3-SO (N = 128)		*P* value
	Mean	SD	Mean	SD	Mean	SD	Mean	SD	
Walking for a quarter mile	1.40	.748	1.46	.814	1.45	.803	1.62	.915	0.002*
Walking up ten steps	1.27	.618	1.31	.656	1.33	.674	1.51	.905	0.001*
Stooping, crouching, kneeling	1.62	.819	1.67	.862	1.73	.893	1.94	1.002	< 0.001*
Lifting or carrying	1.31	.695	1.39	.761	1.45	.816	1.50	.860	< 0.001*
House chore	1.27	.592	1.34	.649	1.34	.652	1.48	.773	< 0.001*
Preparing meals	1.11	.420	1.11	.426	1.10	.394	1.16	.572	0.798
Standing up from armless chair	1.20	.493	1.22	.511	1.25	.563	1.28	.560	0.017*
Getting in and out of bed	1.17	.425	1.19	.468	1.17	.416	1.18	.509	0.967
Standing for long periods	1.58	.881	1.61	.919	1.61	.893	1.80	.991	0.039*
Sitting for long periods	1.33	.641	1.33	.658	1.34	.626	1.38	.710	0.448
Reaching up over head	1.21	.541	1.24	.586	1.25	.575	1.27	.621	0.134
Grasp/holding small objects	1.17	.449	1.20	.502	1.18	.465	1.16	.498	0.274
Going out to movies, events	1.21	.543	1.24	.587	1.22	.562	1.25	.561	0.522
Attending social event	1.20	.536	1.22	.546	1.19	.536	1.17	.534	0.246
Leisure activity at home	1.09	.335	1.11	.379	1.07	.330	1.05	.247	0.026*

Kruskal Wallis Test was used for comparing continuous variables between non-SO and SO groups with different.

*P < 0.05.

**Table 6:** Multivariate model for activity limitations of sarcopenic obesity (SO) and non-sarcopenic obesity (non-SO) participants with different classification after adjusted gender and age.

Difficulty activities	Mean square	F	*P* value
Walking for a quarter mile	4.237	7.300	< 0.001*
Walking up ten steps	3.467	8.674	< 0.001*
Stooping, crouching, kneeling	3.159	4.545	0.003*
Lifting or carrying	0.770	1.481	0.218
House chore	1.783	4.844	0.002*
Preparing meals	0.259	1.471	0.220
Standing up from armless chair	0.254	0.986	0.398
Getting in and out of bed	0.088	0.478	0.698
Standing for long periods	6.181	7.921	< 0.001*
Sitting for long periods	0.503	1.245	0.292
Reaching up over head	0.054	0.175	0.914
Grasp/holding small objects	0.185	0.881	0.450
Going out to movies, events	0.352	1.160	0.323
Attending social event	0.284	0.992	0.395
Leisure activity at home	0.255	2.241	0.081

*P < 0.05.

## Discussion

The present study revealed that individuals with SO experienced more difficulty in performing daily activities relative to those without SO. We ascertained the association between low skeletal muscle mass and varying levels of adiposity and determined the types of physical functional activity impairments associated with SO. Among the ICF-defined daily activities, the activities that required lower limb strength were generally more difficult to perform for the individuals with SO than for those without SO. The findings of the present study can serve as a crucial reference for developing rehabilitation strategies or daily activities performance improvement for patients with SO.

In addition to a rehabilitation exercise program, appropriate diet control is crucial for individuals with SO. A study suggested that to achieve body weight loss, daily caloric intake should be restricted to 500–1000 kcal^21^. The initial diet plan that focuses on achieving a weight loss of 0.5 kg per week may result in an 8–10% reduction in body weight over 6 months. Typically, most individuals can lose approximately 8–10 kg within this timeframe. During the process of losing body weight, maintaining muscle mass is crucial. Methods that enhance protein synthesis during weight loss, such as consuming protein prior to working out or evenly distributing protein intake throughout the day, can prevent sarcopenia caused by weight loss^21^. However, high protein consumption (1.2 g of protein per kg of body weight per day) during a weight loss program may negate the positive effect of weight loss on insulin sensitivity in skeletal muscles^22^. For individuals with SO, distributing protein intake throughout the day or consuming large amounts of protein during main meals can promote muscle protein synthesis^23,24^. Preliminary studies have suggested that combining a protein-rich diet with a weight loss program can enhance physical performance^25^. In a pilot study, participants with SO exhibited improvements in muscle strength and Short Form-36 scores when a high-protein diet was incorporated into their weight loss regimen^26^. Moreover, a recent systemic review recommended higher protein intake with resistance exercise can more effectively reduce the fat mass than exercise alone for SO^27^. These findings indicate that individuals with SO who participate in weight loss programs must maintain sufficient protein intake to combat sarcopenia.

Our study examined the daily functional activity and physical function limitations of individuals with SO, and our findings are consistent with those of other studies. The Concord Health and Aging project was launched to investigate frailty; it involved assessments of adiposity levels and an empirical analysis conducted in accordance with the sarcopenia diagnosis criteria established by the Foundation for the National Institutes of Health. Notably, the project revealed a clear correlation between SO and an escalated risk of frailty. In addition, it identified a relationship between SO and disability, affecting both activities of daily living and instrumental activities of daily living^28^. Baumgartner et al.^9^ compared healthy individuals and individuals with SO over an 8-year period and demonstrated that SO is a risk factor for disability. They also explored the transition from having obesity with low muscle mass to a having a healthy body composition, thereby providing insightful findings regarding functional mobility. These findings provide the foundation for understanding the implications of the interplay between obesity, sarcopenia, and disability in activities of daily living. For quality of life, few studies have explored the effect of SO on quality of life. A study reported a link between SO and unfavorable results on the Medical Outcomes Survey^29^. In the future, researchers should focus on health-related quality of life and patient-reported outcomes in the context of SO to clarify the rehabilitation needs of individuals with SO.

Through the examination of NHANES data, our study highlighted the activity limitations of people with SO. Nevertheless, it has several limitations that should be addressed. First, the NHANES database collected cross-sectional data, which can only demonstrate the associations of increased adiposity with physical functional impairment. Therefore, the causality of adiposity and physical functional activity limitations could not be clearly identified. Additional longitudinal investigations are required to furnish more evidence regarding causality in the future. Second, relying solely on self-reports may introduce biases and compromise the accuracy of assessments related to physical functioning, as individuals may not provide precise representations of their actual capabilities. Integrating self-reports with objective measures, such as performance-based tests or biomechanical assessments, would have augmented the validity and reliability of the comprehensive assessment. The muscle strength-related parameters, such as grip strength and muscle architecture, were not included in this study^30^. The association between different body composition classifications of SO and muscle strength cannot be presented in this study. Third, DXA-related limitations should be acknowledged, including the exclusion of patients heavier than 136 kg or taller than 196 cm. Additionally, assumptions of constant hydration and tissue density that are made in DXA must be considered when applying the proposed ASMI and FMI thresholds. The DXA data from the NHANES were obtained using Hologic instruments and have been adjusted as per the suggestions of Schoeller et al.; consequently, the generalizability of the data to the equipment of other manufacturers is limited^16^. Fourth, the survey only evaluated noninstitutionalized older adults; that is, older adults with severe sarcopenia or obesity, such as nursing home residents or individuals with severe disabilities, were excluded. Consequently, the actual prevalence in these subgroups was probably underestimated^31^. Fifth, employing cutoffs for classifying individuals might not effectively capture the continuous nature of physiological parameters, potentially oversimplifying intricate relationships. Conducting secondary analyses on a subgroup with fewer overlaps could be beneficial. This approach might yield a more nuanced understanding by exploring a subgroup with clearer boundaries, potentially mitigating the influence of minor variations on classification. This strategy has the potential to refine the precision of findings and provide insights into the distinctive characteristics of individuals within a more precisely defined range of body fat percentages. Finally, when contemplating a larger sample size across various SO classification analyses, the racial differences were not factored into this study. This aspect should be acknowledged as a potential confounder for subsequent investigations in the future.

## Conclusion

In our investigation, we established a correlation between diminished skeletal muscle mass and diverse adiposity levels, elucidating the specific physical functional activity impediments linked to sarcopenic obesity (SO). A heightened degree of SO was found to correspond with increased challenges in executing routine daily tasks, especially those reliant on lower limb strength.

### Supplementary Information

Supplementary Table S1.

Supplementary Table S2.

Supplementary Table S3.

Supplementary Table S4.

## Data Availability

The data will be shared on reasonable request to the corresponding author.
