# Supplementary material for: Association of physical functional activity impairment with severity of sarcopenic obesity: findings from National Health and Nutrition Examination Survey
Source: Sci Rep. 2024 Feb 15;14:3787. doi: 10.1038/s41598-024-54102-z (PMC10869697; doi:10.1038/s41598-024-54102-z)
Supplement: Supplementary file 1 — Supplementary Table S1. [file 41598_2024_54102_MOESM1_ESM.docx]

Table 1 Demographic and body composition characteristics of sarcopenic obesity (SO) and non-sarcopenic obesity (non-SO) participants for Female

|  | Non-SO (N=1620) | | SO (N=2075) | | P-value |
| --- | --- | --- | --- | --- | --- |
|  | Mean | SD | Mean | SD |  |
| Age | 53.83 | 17.32 | 63.03 | 14.63 | <0.001 |
| Total Area (cm^2^) | 1918.55 | 212.90 | 1833.40 | 169.67 | <0.001 |
| Total BMC (g/cm^2^) | 1.07 | 0.12 | 1.01 | 0.12 | <0.001 |
| Total Fat (g) | 34505.83 | 15915.19 | 29984.13 | 6449.71 | <0.001 |
| Total Lean excl BMC (g) | 45475.01 | 9727.37 | 38510.27 | 4800.38 | <0.001 |
| Total Lean+Fat (g) | 82047.96 | 25013.61 | 70360.54 | 10214.46 | <0.001 |
| Total Percent Fat | 40.05 | 7.82 | 42.32 | 4.10 | <0.001 |
| Weight (kg) | 81.55 | 25.00 | 69.86 | 10.22 | <0.001 |
| Standing Height (cm) | 161.00 | 7.08 | 159.00 | 7.04 | <0.001 |
| BMI (kg/m^2^) | 31.39 | 9.24 | 27.61 | 3.51 | <0.001 |
| ASMI | 7.50 | 1.68 | 6.16 | 0.67 | <0.001 |
| FMI | 13.29 | 6.03 | 11.86 | 2.46 | <0.001 |

Chi-square analysis was used for comparing categorial variables between non-SO and SO groups with different classification

ANOVA was used for comparing continuous variables between non-SO and SO groups with different classification

BMC, bone mineral density; BMI, body mass index; ASMI, appendicular skeletal muscle mass index; FMI, fat mass index
