# Supplementary material for: Association of physical functional activity impairment with severity of sarcopenic obesity: findings from National Health and Nutrition Examination Survey
Source: Sci Rep. 2024 Feb 15;14:3787. doi: 10.1038/s41598-024-54102-z (PMC10869697; doi:10.1038/s41598-024-54102-z)
Supplement: Supplementary file 2 — Supplementary Table S2. [file 41598_2024_54102_MOESM2_ESM.docx]

Table 1 Demographic and body composition characteristics of sarcopenic obesity (SO) and non-sarcopenic obesity (non-SO) participants for Male

|  | Non-SO (N=3287) | | SO (N=179) | | P-value |
| --- | --- | --- | --- | --- | --- |
|  | Mean | SD | Mean | SD |  |
| Age | 58.66 | 16.37 | 72.09 | 11.20 | <0.001 |
| Total Area (cm^2^) | 2234.78 | 206.37 | 2131.53 | 182.94 | <0.001 |
| Total BMC (g/cm^2^) | 1.16 | 0.12 | 1.09 | 0.12 | <0.001 |
| Total Fat (g) | 25736.53 | 10000.17 | 27882.69 | 4163.85 | 0.004 |
| Total Lean excl BMC (g) | 57789.18 | 9940.35 | 48612.77 | 5231.33 | <0.001 |
| Total Lean+Fat (g) | 86128.67 | 18797.08 | 78837.87 | 8682.69 | <0.001 |
| Total Percent Fat | 29.03 | 5.82 | 35.31 | 2.71 | <0.001 |
| Weight (kg) | 85.55 | 18.69 | 78.51 | 8.51 | <0.001 |
| Standing Height (cm) | 173.73 | 7.60 | 170.89 | 7.65 | <0.001 |
| BMI (kg/m^2^) | 28.26 | 5.49 | 26.84 | 1.62 | <0.001 |
| ASMI | 8.38 | 1.34 | 6.79 | 0.45 | <0.001 |
| FMI | 8.50 | 3.17 | 9.53 | 1.13 | <0.001 |

Chi-square analysis was used for comparing categorial variables between non-SO and SO groups with different classification

ANOVA was used for comparing continuous variables between non-SO and SO groups with different classification

BMC, bone mineral density; BMI, body mass index; ASMI, appendicular skeletal muscle mass index; FMI, fat mass index
