# Supplementary material for: Association of physical functional activity impairment with severity of sarcopenic obesity: findings from National Health and Nutrition Examination Survey
Source: Sci Rep. 2024 Feb 15;14:3787. doi: 10.1038/s41598-024-54102-z (PMC10869697; doi:10.1038/s41598-024-54102-z)
Supplement: Supplementary file 3 — Supplementary Table S3. [file 41598_2024_54102_MOESM3_ESM.docx]

Table 2 Demographic and body composition characteristics of sarcopenic obesity (SO) and non-sarcopenic obesity (non-SO) participants with different severity for Female

|  | Non-SO (N=1620) | | classes 1-SO  (N=689) | | classes 2-SO  (N=1258) | | classes 3-SO  (N=128) | | P-value |
| --- | --- | --- | --- | --- | --- | --- | --- | --- | --- |
|  | Mean | SD | Mean | SD | Mean | SD | Mean | SD |  |
| Age | 53.83 | 17.32 | 59.65 | 15.58 | 64.32 | 13.94 | 68.59 | 12.05 | <0.001 |
| Total Area (cm^2^) | 1918.55 | 212.90 | 1854.10 | 180.52 | 1822.81 | 164.46 | 1825.98 | 149.61 | <0.001 |
| Total BMD (g/cm^2^) | 1.07 | 0.12 | 1.03 | 0.12 | 1.00 | 0.12 | 0.98 | 0.10 | <0.001 |
| Total Fat (g) | 34505.83 | 15915.19 | 29602.79 | 8270.37 | 29825.57 | 5323.66 | 33595.15 | 3755.53 | <0.001 |
| Total Lean excl BMC (g) | 45475.01 | 9727.37 | 40263.40 | 5369.89 | 37853.86 | 4248.42 | 35524.68 | 3396.54 | <0.001 |
| Total Lean+Fat (g) | 82047.96 | 25013.61 | 71790.22 | 12742.60 | 69521.37 | 8764.44 | 70912.44 | 6750.85 | <0.001 |
| Total Percent Fat | 40.05 | 7.82 | 40.64 | 4.75 | 42.72 | 3.27 | 47.34 | 1.99 | <0.001 |
| Weight (kg) | 81.55 | 25.00 | 71.28 | 12.75 | 69.03 | 8.76 | 70.42 | 6.78 | <0.001 |
| Standing Height (cm) | 161.00 | 7.08 | 159.68 | 7.15 | 158.71 | 7.04 | 158.12 | 6.17 | <0.001 |
| BMI (kg/m^2^) | 31.39 | 9.24 | 27.92 | 4.57 | 27.39 | 2.92 | 28.09 | 1.59 | <0.001 |
| ASMI | 7.50 | 1.68 | 6.53 | 0.74 | 6.03 | 0.55 | 5.48 | 0.34 | <0.001 |
| FMI | 13.29 | 6.03 | 11.61 | 3.18 | 11.84 | 2.02 | 13.41 | 1.00 | <0.001 |

Chi-square analysis was used for comparing categorial variables between non-SO and SO groups

Independent t test was used for comparing continuous variables between non-SO and SO groups

BMC, bone mineral density; BMI, body mass index; ASMI, appendicular skeletal muscle mass index; FMI, fat mass index
