# Supplementary material for: Association of physical functional activity impairment with severity of sarcopenic obesity: findings from National Health and Nutrition Examination Survey
Source: Sci Rep. 2024 Feb 15;14:3787. doi: 10.1038/s41598-024-54102-z (PMC10869697; doi:10.1038/s41598-024-54102-z)
Supplement: Supplementary file 4 — Supplementary Table S4. [file 41598_2024_54102_MOESM4_ESM.docx]

Table 2 Demographic and body composition characteristics of sarcopenic obesity (SO) and non-sarcopenic obesity (non-SO) participants with different severity for Male

|  | Non-SO (N=3287) | | classes 1-SO  (N=137) | | classes 2-SO  (N=42) | | P-value |
| --- | --- | --- | --- | --- | --- | --- | --- |
|  | Mean | SD | Mean | SD | Mean | SD |  |
| Age | 58.66 | 16.37 | 72.10 | 11.15 | 72.07 | 11.52 | <0.001 |
| Total Area (cm^2^) | 2234.78 | 206.37 | 2149.66 | 169.58 | 2072.42 | 212.68 | <0.001 |
| Total BMD (g/cm^2^) | 1.16 | 0.12 | 1.10 | 0.12 | 1.06 | 0.14 | <0.001 |
| Total Fat (g) | 25736.53 | 10000.17 | 27520.95 | 4386.02 | 29062.67 | 3097.78 | 0.011 |
| Total Lean excl BMC (g) | 57789.18 | 9940.35 | 49373.66 | 4791.29 | 46130.82 | 5866.76 | <0.001 |
| Total Lean+Fat (g) | 86128.67 | 18797.08 | 79274.89 | 8633.92 | 77412.38 | 8792.57 | <0.001 |
| Total Percent Fat | 29.03 | 5.82 | 34.60 | 2.45 | 37.64 | 2.16 | <0.001 |
| Weight (kg) | 85.55 | 18.69 | 78.84 | 8.56 | 77.45 | 8.33 | <0.001 |
| Standing Height (cm) | 173.73 | 7.60 | 171.17 | 7.59 | 169.98 | 7.85 | <0.001 |
| BMI (kg/m^2^) | 28.26 | 5.49 | 26.87 | 1.76 | 26.76 | 1.07 | 0.003 |
| ASMI | 8.38 | 1.34 | 6.91 | 0.37 | 6.38 | 0.48 | <0.001 |
| FMI | 8.50 | 3.17 | 9.37 | 1.20 | 10.05 | 0.66 | <0.001 |

Chi-square analysis was used for comparing categorial variables between non-SO and SO groups

Independent t test was used for comparing continuous variables between non-SO and SO groups

BMC, bone mineral density; BMI, body mass index; ASMI, appendicular skeletal muscle mass index; FMI, fat mass index
